# Relationship between oral health and Fried’s frailty criteria in community-dwelling older persons

**DOI:** 10.1186/s12877-017-0568-3

**Published:** 2017-08-01

**Authors:** Benedikta Kamdem, Laurence Seematter-Bagnoud, Fabiana Botrugno, Brigitte Santos-Eggimann

**Affiliations:** Health Services Unit, Institute of Social and Preventive Medicine (IUMSP), Route de la Corniche 10, 1010 Lausanne, CH Switzerland

**Keywords:** Frailty, Oral health, Oral pain, Chewing problems, Masticatory ability

## Abstract

**Background:**

Oral health and frailty might be linked through several pathways, but previous studies are scarce. This study examined the association between oral health and components of Fried’s frailty phenotype.

**Methods:**

This cross-sectional analysis was based on a sample of 992 community-dwelling persons aged 73 to 77 years observed in the 2011 follow-up of the Lausanne 65+ cohort (Lc65+) study. Data were collected through annual mailed questionnaires, interview and physical examination. Oral health was assessed according to self-reported oral pain and masticatory ability. Frailty was defined as meeting at least one criterion of the Fried’s phenotype.

**Results:**

Oral pain was reported by 14.8% and chewing problems by 9.7%. Impaired masticatory ability (IMA) was more frequent in subjects with missing teeth or removable dentures (13.5%) than among those with full dentition or fixed dental prostheses (3.2%). In logistic regression analyses adjusting for demographics, alcohol consumption, smoking, comorbidity and financial difficulties, persons with oral pain and those with chewing problems had significantly higher odds of being frail (adjusted OR_pain_ = 1.72; 95% CI 1.17–2.53 and adjOR_IMA_1.70; 1.07–2.72, respectively). Lack of endurance was associated with both oral pain (adjOR = 3.61; 1.92–6.76) and impaired masticatory ability (adjOR = 2.20; 1.03–4.72). The latter was additionally linked to low physical activity (adjOR = 2.35; 1.29–4.28) and low gait speed (adjOR = 3.12; 1.41–6.90), whereas oral pain was associated with weight loss (adjOR = 1.80; 1.09–2.96) and low handgrip strength (adjOR = 1.80; 1.17–2.77).

**Conclusion:**

Self-reported oral pain and chewing impairment had a significant relation with frailty and its components, not only through a nutritional pathway of involuntary weight loss. Longitudinal analyses are needed to examine whether a poor oral condition might be a risk factor for the onset of frailty.

## Background

Oral health has both direct and indirect effects on seniors’ health [1]. With advancing age, oral changes occur, and may lead to reduced salivary flow rate and altered sense of taste. These changes are risk factors for a variety of oral problems such as tooth loss, and periodontal diseases [2] that may finally lead to tooth loss and edentulism through chronic inflammation. Edentulism as well as the use of nonfunctional dentures often causes chewing problems, which in turn may lead to food selection and unbalanced diet in older adults [3]. Furthermore, it is recognized that the chronic inflammation caused by a poor periodontal status is a risk factor for cardiovascular disease, which is a major determinant of older persons’ health, functional status, and frailty [4, 5]. Frailty syndrome is characterized by decreased physiological reserves, and increased vulnerability to adverse events. It has been associated with an increased risk for disability, falls, hospitalization and death [6]. According to Fried’s definition of the frailty phenotype, frail persons are identified as having three or more of the five followings components: unintentional weight loss, poor endurance and energy, low physical activity, slowness, and weakness. Pre-frail have one or two criteria, while persons having none of them are considered robust.

To investigate the link between oral health and frailty, previous studies often focused on the association between one frailty criterion and a specific oral problem. Moreover, as illustrated in a recent literature review [7], they often applied different oral health measures and frailty’s criteria.

The clearest relationship, widely addressed in the literature, is mediated through nutrition [8]. The evidence indicates that individuals with few teeth or with non-functional dentures have inadequate nutrient intake, because they avoid certain foods, and are more likely to lose weight involuntarily [3, 9, 10].

Besides, several authors explored the relationship between chewing ability as well as dental occlusion, with low physical activity, weakness and slowness. These associations are likely mediated through muscle strength and body balance [11–14]. Indeed, poor chewing function, self-reported or measured by professionals, is significantly linked to reduced daily physical activity [15]. In otherwise healthy older people, poor dental occlusion may also be a predictor for a decline in physical fitness. Notably, it has been associated with a decline in lower extremity strength, and gait speed [16]. Another condition examined is loss of teeth which has been identified as a risk factor for the onset of fatigue in older persons, aged 70 to 80 years, although this association disappeared after adjustment for confounders [17].

Only three cross-sectional analyses examined the association between oral health and Fried’s frailty phenotype. Their results suggested that having less than 21 teeth, the need of fixed or removable dental prostheses, non-functional dentures as well as poor utilization of dental services and poorer self-perception of oral health, were associated with a higher probability of being frail [5, 9, 18].

The aim of this study was to consider the link between oral health, more specifically pain and impaired masticatory ability, and each component of Fried’s frailty phenotype.

## Methods

### Study population

The study participants are women and men aged 65 and older, living in the community, enrolled in the Lausanne cohort 65+ study [19]. This longitudinal study aims to provide a better understanding of risk factors, manifestations and consequences of frailty.

Briefly, the participants aged 65 to 70 years were randomly selected from the Lausanne city’s population. Subjects living in an institution or unable to respond by themselves were excluded. Thus, among 3′056 eligible persons, 1′564 were enrolled into the study in 2004. Based on a comparison with data from the 2000 Swiss national population census, participants were representative of the Lausanne general population in the same age category regarding nationality, marital status, place of birth, living arrangement, and professional activity [19].

Data were collected using annual self-administered mailed questionnaires, complemented by triennial face-to-face interviews with physical examination and physical and cognitive performance tests.

Data about demographics, education level, smoking history, alcohol consumption, falls, hospitalizations, current chronic diseases and number of chronic diseases treated with drugs, were collected using standardized questionnaires. Interviews and performance tests were carried out according to a standardized protocol by trained and calibrated medical assistants. Gait speed was measured over a 20-m well-lighted walkway with participants walking at self-selected speed. Handgrip strength was measured by hydraulic manometer (Seahan®).

In 2011, a set of questions on oral health were added to the interview questionnaire, which was attended by 1006 (71.7% of survivors from the initial sample) participants. Among them, 992 participants having undergone frailty assessment were included in this secondary data analysis.

### Variables

#### Frailty

Frailty was measured according to the five criteria of Fried’s phenotype [19]:

- Low muscle strength: cut-off for low handgrip strength used in Fried et al.

- Poor nutrition: self-reported unintentional weight loss during last 12 months.

- Poor endurance: self-reported lack of energy and fatigue during last 4 weeks.

- Slow walking: cut-off for low gait speed used in Fried et al.

- Low physical activity: defined as doing less than 20 min of physical exercise per week, and walking less than 90 min per week. Participants fulfilling these criteria were nevertheless considered active if they reported a high amount of daily usual physical activity such as climbing stairs, or lifting weights.

The outcome was meeting at least one frailty criterion. Indeed, the prevalence of frailty as defined by meeting at least three frailty criteria was less than 5%, precluding statistical analyses comparing frail to pre-frail individuals, i.e. having one or two frailty criteria.

#### Oral health variables

Several variables were operationalized based on the questions about oral health. To evaluate dental status, the following two questions were asked: “Do you still have your natural teeth?”, “How your missing teeth were replaced?”. Four categories were defined: full dentition, missing teeth replaced by fixed dental prostheses or by removable dentures or not replaced at all. Oral health was assessed through two items. Oral pain was defined as a positive answer (“yes, mildly” or “yes, a lot” vs “not at all”) to the question: “Do you have any pain or sensitivity on gums or teeth while chewing?”. Impaired masticatory ability (IMA) was based on the question: “Are you able to chew all types of food?” and was positive when the answer was “yes, but hardly” or “no, I swallow whole” vs “yes, without difficulty”.

#### Covariates

The co-variables were demographic factors (age, gender), number of chronic diseases based on a pre-defined list of ten (diabetes mellitus, heart disease, respiratory illnesses, cancer, osteoporosis, osteoarthritis, ulcer, stroke, hypertension and depression), cognitive impairment (defined as a score of <24 at the Mini-Mental State Examination), smoking status, alcohol consumption during the previous 12 months (moderate drinking was defined as at maximum 1 units per day in women and 2 units per day in men, and no binge drinking), education (highest level completed) and financial situation. Financial problems were defined as having trouble making ends meet over the last 12 months or being exempted from paying compulsory health insurance based on income declaration.

### Statistical analysis

The distribution of participants’ characteristics was described using frequencies (categorical variables) or arithmetic means and standard deviations (continuous variables). The association between dental status and the presence of pain or chewing problems was examined using a chi-square test.

A first set of logistic regression models examined the odds of being frail, using oral pain and IMA as explanatory variables, respectively.

Each model was successively and additionally adjusted for 1) age and gender, 2) chronic diseases and cognitive impairment 3) smoking status and alcohol consumption 4) education status and financial problems.

Then, regression models were performed using each of the five Fried’s frailty criteria as dependent variables, and each of the oral health indicators as explanatory variables. The same hierarchical adjustment was used as described above. The presence of an interaction between oral pain and IMA on frailty was examined. The level of significance used in this study was *P* < 0.05.

## Results

Table 1 summarizes the characteristics of the population. The mean age was 74.9 years (range 73–77). Sixty percent of the participants were female. One in five persons had only compulsory school education; approximately 40% had two chronic diseases or more, while a quarter of the sample showed symptoms of depression. In all, 9% scored below the cut-off of the cognition test (MMSE <24). Thirteen percent were current smokers and one of four reported risky alcohol consumption. Financial problems were reported by 12.3% of the sample. About a third of the participants presented at least with one frailty criterion (35.4%).

**Table 1:** Characteristics of the study participants (*N* = 992)

Characteristic		Proportion (%)
Age (years, mean+/− SD)	74.9 (+/−1.39)	
Gender		
	Female	59.8
Education level		
	Compulsory school	22.8
	Apprenticeship/high school	64.4
	Tertiary education	12.7
Financial problems		
	Yes	12.3
Chronic diseases^a^		
	0	24.8
	1	33.6
	≥ 2	41.6
Cognitive impairment		
	MMSE <24	9.0
Alcohol consumption		
	At risk	25.9
	Moderate	67.4
	No drinking	6.6
Smoking status		
	Yes	13.0
	Stopped since <35y	25.1
	Stopped since >35y	12.6
	Never	48.5
Dental Status		
	No missing tooth	5.4
	Missing teeth, fixed dentures	32.1
	Missing teeth, removable dentures	41.0
	Missing teeth, no dentures	21.5

^a^Number of diseases self-reported out of the following list: hypertension, heart diseases, stroke, diabetes mellitus, cancer, chronic respiratory disease, arthritis, osteoporosis, gastro-intestinal diseases, depression, neurodegenerative diseases

**Fig. 1:**
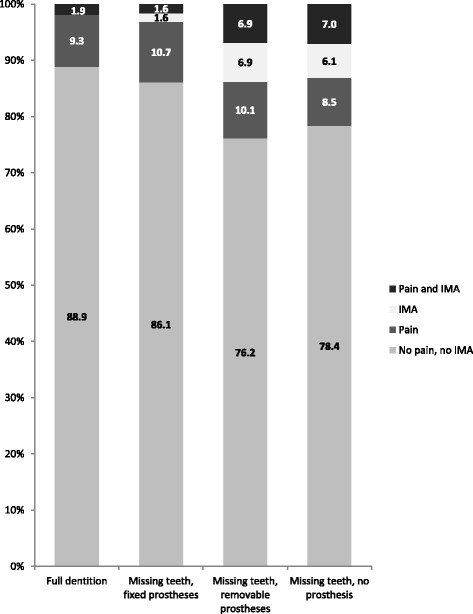
Prevalence of oral pain and impaired masticatory ability (IMA) according to dental status

Regarding dental status, a small minority (5%) stated a full dentition, 73% wore removable dentures or fixed dental prostheses, while missing teeth were not replaced in about 20%. Participants with full dentition or wearing fixed dental prostheses had significantly lower prevalence of chewing problems (alone or with pain 3.2%) than those whose missing teeth were replaced by removable dentures or not replaced at all (13.5%, Fig. 1). Interestingly, there was no association between dental status and oral pain alone, with a prevalence reaching about 10% in each category.

Based on regression analysis adjusting for age and gender, both oral pain and IMA were significantly associated with the presence of one or more frailty criteria. These associations remained after successive adjustment for all other covariates (adjOR_pain_ = 1.72; 95% CI 1.17–2.53 and adjOR_IMA_ = 1.70; CI 1.07–2.72, Table 2). The interaction between pain and IMA was tested and came out to be not significant.

**Table 2:** Results of the logistic regression models on the association between oral health problems and components of the frailty phenotype

	Oral pain		Impaired masticatory ability	
	Sex- and age-adj.*OR [95%CI] *p*-value	Fully adj.**OR [95%CI] *p*-value	Sex- and age-adj.*OR [95%CI] *p*-value	Fully adj.**OR [95%CI] *p*-value
Frailty phenotype (+1 criterion)	1.85 [1.29–2.65] < .01	1.72 [1.17–2.53] < .01	1.90 [1.24–2.92] < .01	1.70 [1.07–2.72] .02
Weight loss	1.87 [1.16–3.01] .01	1.80 [1.09–2.96] .02	1.67 [0.94–2.96] > .05	1.61 [0.89–2.95] > .05
Lack of endurance	3.30 [1.84–5.90] < .01	3.61 [1.92–6.76] < .01	2.27 [1.12–4.60] .02	2.20 [1.03–4.72] .04
Low physical activity	1.62 [0.97–2.71] > .05	1.65 [0.90–2.68] > .05	2.58 [1.48–4.49] < .01	2.35 [1.29–4.28] < .01
Low handgrip strength	1.93 [1.30–2.88] < .01	1.80 [1.17–2.77] < .01	1.73 [1.08–2.79] .02	1.51 [0.89–2.54] > .05
Low gait speed	1.26 [0.59–2.67] > .05	1.12 [0.49–2.57] > .05	2.84 [1.38–5.85] < .01	3.12 [1.41–6.90] < .01

*Adjustment for sex and age only

**After adjustment for age and gender, as well as number of chronic diseases (0; 1;>2), cognitive impairment (MMSE <24), smoking status (never; past; current), alcoholic consumption (no drinking; moderate, at risk), education and financial problems

The relationship between both oral pain and IMA with each frailty criterion was positive, as the results from the logistic regression models indicated that all odds ratios were above 1.00. However, some criteria showed a significant relationship with only pain and not IMA, and vice-versa, while lack of endurance was significantly associated with both (adjOR_pain_ = 3.61; 1.92–6.76, and adjOR_IMA_ = 2.20; 1.03–4.72). Participants who reported oral pain were significantly more likely to report involuntary weight loss (adjOR_pain_ = 1.80; 1.09–2.96) and to have low handgrip strength (adjOR_pain_ = 1.80; 1.17–2.77). In contrast, the participants with IMA had greater odds of having a low physical activity (adjOR_IMA_ = 2.35; 1.29–4.28) and a low gait speed (adjOR_IMA_ = 3.12; 1.41–6.90)*.* As to low handgrip strength, its association with reported chewing problems was no longer significant after adjustment for comorbidity and cognitive impairment.

## Discussion

This study found significant associations between self-reported impaired masticatory ability and oral pain with the Fried’s frailty phenotype. As a distinguishing feature, it examined the association between oral health and each frailty criterion. After adjustment for potential confounders, impaired masticatory ability was significantly associated with low physical activity and low gait speed, whereas oral pain was linked with weight loss and low handgrip strength. Both were associated with lack of endurance.

### Oral health and frailty

Two previous studies, conducted among community-dwelling older persons in South America [5, 18] observed a cross-sectional association between oral health problems and Fried’s frailty phenotype, even when adjusted for socio-demographics, general health and the number of remaining teeth, among other potential confounders. One focused on a poor self-perception of oral health as compared to others of the same age [5], while the other relied on the need for dental prosthesis based on a dentist’s examination (i.e. poorly adjusted prosthesis or non-prosthesis) [18]. Altogether, these results and those of the current study meet to suggest that significant oral health problems, self-reported or objectively measured, might be a marker for frailty, although the cross-sectional nature of the analyses precludes further investigating a potential causal effect.

### Chewing problems and frailty criteria

In our sample, people with missing teeth replaced by a removable denture or not replaced were more likely to have impaired mastication, suggesting that replacement of missing teeth by removable dentures might not alleviate the functional shortcomings of tooth loss and hence ensure optimal chewing function [20]. This study indicates an independent association of impaired masticatory ability with a low level of physical activity and a slow gait. These observations should be considered in the context of frailty prevention, as masticatory ability could be linked to frailty through different pathways. First, several studies indicate that a poor dental support, which may be considered a surrogate for impaired mastication, is associated with abnormal body posture, poor balance and more generally with a decline in physical fitness and strength [12, 16, 21].

Additionally, a longitudinal study [11] found that a poor dental occlusion did lead to a decreased leg extension power, which in turn generated a loss of balance and a reduction in gait speed. It is plausible that this decrease in physical performance progressively causes a reduction in usual activities, which might support the association between impaired mastication and low physical activity observed in this study.

As another mechanism, some studies showed that dental status and chewing ability are significantly related to nutritional intake and nutritional status [9, 10], but the association with weight loss is not well established [22, 23]. Since a decline in nutritional intake may lead to involuntary weight loss, it can be expected that impaired masticatory performance may be related to weight loss [24]. Nevertheless, the absence of significant association between impaired mastication and involuntary weight loss in the present study does not support this hypothesis. The changes in diet following tooth loss most frequently refer to a selection of foodstuffs that are easy to chew [25]. Depending on the cultural context and the level of education, calories will be obtained from high level of sugar and refined carbohydrates convenience food, hence a less healthy food selection [26]. These results are consistent with other research works [27], and might be explained by the fact that it is possible to swallow, without chewing, highly calorific foods. Unfortunately, data on the diet of the participants do not allow further investigating this hypothesis. Finally, there was a significant age- and gender-adjusted association with low handgrip strength, which did not remain significant after further adjustment for comorbidity and cognitive impairment. Moriya’s research observed a significant relationship between handgrip strength and self-assessed masticatory ability, after taking comorbidity into account [12]. Residual confounding might be an explanation for these Moryia’s divergent findings, as his study did not adjust for cognitive impairment. More studies, preferably longitudinal, are needed to better understand the relationship between masticatory performance and weight changes as well as in older adults.

### Oral pain and frailty criteria

One study suggested that oral pain and discomfort are significantly correlated with handgrip strength, even when taking account of demographic factors, psychosocial, medical and dental status [13]. Our findings are in agreement with those of that study where oral pain and handgrip strength showed a significant negative correlation. Hence, oral pain and discomfort might influence muscle strength of the upper limbs, maybe indirectly, via a negatively affecting the chewing ability [12]. Cognitive impairment has been shown to negatively influence chewing ability [14], while having less effect on oral pain. Though we cannot exclude the link between impaired mastication and handgrip strength, oral pain has a stronger relationship to muscle strength in this study. As another process contributing to the association between pain and poor handgrip strength, oral pain might be caused by chronic inflammation, which may be associated with decline in strength [28]. Interestingly, oral pain was found to be significantly and independently associated to involuntary weight loss, suggesting that oral pain may lead to insufficient caloric intake in a greater dimension than chewing efficiency [8].

Further, lack of endurance was more likely in older persons reporting oral pain. It is worth noting that the link between oral health and endurance, as a component of Fried’s phenotype, has not been explored very often. A prospective study [17] examined the role of tooth loss in the onset of fatigue after the age of 70 years, and found that the observed association disappeared after adjusting for smoking status, comorbidity and socio-economic position. Our study highlights that self-reported oral pain or discomfort might indeed represent a risk factor for fatigue in the elderly population. A longitudinal study would be needed to confirm this hypothesis.

### Study limitations and strength

This study has some limitations. First, because of the cross-sectional nature of the study, we cannot fully examine the causal relationship between oral pain or impaired masticatory ability and the incidence of frailty and its components. Then, oral condition is ideally obtained through an oral examination, and chewing performance objectively measured using sieving methods [29] or assessing the degree of color mixture from two-colored chewing specimen [30]. In this study, dental status and oral health problems were self-reported. Subjects reported whether or not they still had all their natural teeth, without indicating the number of missing teeth, and subjectively evaluated their ability to chew food. Yet, previous studies showed that perception of poor oral health and pain and chewing problems were associated to frailty or its components, while it was not always the case with objective indicators such as the number of teeth.

The potential effect of medication on oral health, as a consequence of hyposalivation, could not be examined, due to limited information on medication use.

Finally, one could doubt about the reliability of the information about oral health reported by the participants who had abnormal cognitive test, and represented about 9% of the sample. As the questions on dental health were part of the face-to-face interview, participants who had difficulty understanding the question were helped by the medical assistant to understand the question. Besides, participants with severe cognitive impairment are followed-up with questionnaires filled by proxies and do not come for the in-person visit.

However, as a major strength, this analysis was undertaken on a large sample of older persons who were recruited from the community and not from dental practices, enhancing the generalizability of the results. Further, the evaluation of frailty and general health were based on personal interview and physical examinations performed by trained medical assistants. In addition, the large range of data collected allowed adjusting the analyses for demographics, comorbidity, cognitive impairment as well as for smoking status, alcohol consumption and financial problems.

## Conclusion

In conclusion, significant relationships were found between self-reported oral pain, perceived chewing impairment and components of the frailty phenotype in older adults. While lack of endurance was significantly associated with both types of oral problems, weight loss and low handgrip strength were significantly linked to oral pain whereas low physical activity and low gait speed were significantly related to chewing problems. All associations remained after adjusting for covariates. Our findings suggest that oral health condition might be an important factor in the onset of frailty, not only through a nutritional pathway of involuntary weight loss. However, longitudinal studies are needed to investigate whether there is a causal relationship. In view of an aging population, our findings could have implications in health policy. Indeed, those results may increase awareness for health care professionals, including geriatric and dental care providers, of the dental care needs of older adults, since a poor oral condition might indicate a transition towards frailty.

## Abbreviation

IMA: Impaired masticatory ability
